# Analysis of oxidative stress, inflammation and endothelial function following intravenous iron in chronic kidney disease in the Iron and Heart Trial

**DOI:** 10.1038/s41598-022-10717-8

**Published:** 2022-04-27

**Authors:** Xenophon Kassianides, Victoria Allgar, Iain C. Macdougall, Philip A. Kalra, Sunil Bhandari

**Affiliations:** 1 Academic Renal Research Department, Hull University Teaching Hospitals NHS Trust and the Hull York Medical School, Kingston upon Hull, UK; 2 PenCTU, Peninsula Medical School (Faculty of Heath), Plymouth, UK; 3 Department of Renal Medicine, King’s College Hospital, London, UK; 4 Department of Renal Medicine, Salford Royal NHS Foundation Trust and University of Manchester, Manchester, UK

**Keywords:** Kidney, Kidney diseases, Chronic kidney disease, Anaemia, Inflammation

## Abstract

Iron deficiency commonly affects patients with chronic kidney disease and has an important burden in disease trajectory and quality of life; nonetheless current guidelines do not advocate treatment of iron-deficiency without anemia in this patient group. Concerns exist regarding the potential effects of intravenous iron on oxidative stress, inflammation, and endothelial function. As part of a multicenter double-blinded randomized controlled clinical trial, we examined the effects of a single dose of intravenous iron vs. placebo on biomarkers of oxidative stress, inflammation and endothelial function in non-anemic iron deficient patients (serum ferritin < 100 μg/L and/or transferrin saturation < 20%) with chronic kidney disease (stage 3b-5). Fifty-four individuals were randomized to receive ferric derisomaltose (n = 26) or placebo (n = 28). Ferric derisomaltose was associated with a non-significant decrease in mean F2-isoprostane and no effect on thiobarbituric acid reactive substances when compared to placebo throughout follow up. No effect on inflammatory markers was observed. A modest but statistically significant rise in E-selectin was noted in the intravenous iron group at 1 month and 3 month follow-up (p = 0.030 and p = 0.002 respectively). These results suggest ferric derisomaltose administration in non-dialysis dependent chronic kidney disease patients who are iron deficient does not induce prolonged oxidative stress or inflammation. Larger trials are required to quantify the benefit of intravenous iron administration in this patient group.

## Introduction

Iron deficiency frequently occurs in patients with non-dialysis dependent chronic kidney disease (ND-CKD)^1^. It is associated with worsening clinical outcomes and quality of life, and its alleviation may be key in improving the lives of people with CKD^2^. Current guidelines advocate for the correction of anemia using iron (oral/intravenous (IV)) in this patient group^3^; however, the evidence on alleviation of iron deficiency without anemia is scarce. Studies in heart failure have revealed an improvement in functional status following supplementation with IV iron in terms of 6-min walking distance, New York heart association dyspnea scale and patient reported outcome measures, whilst evidence from the use of a proactive high-dose IV iron approach in patients on hemodialysis was positive in relation to cardiovascular outcomes^4,5^. Similarly, high-dose iron supplementation with modern IV iron preparations (ferric derisomaltose) in ND-CKD patients has been shown to have a reduced incidence of cardiovascular events when compared to older preparations (iron sucrose) given in smaller doses^6^. The potential underlying beneficial mechanism of treatment of non-anemic iron deficient CKD patients with high dose IV iron is unclear, and indeed studies have shown variable impact on oxidative stress, inflammation and endothelial function in both non-dialysis and dialysis-dependent CKD patients following administration of iron^7–9^.

One persisting concern regarding the use of IV iron in the ND-CKD population is the potential propensity for oxidative stress secondary to non-transferrin bound iron (NTBI) being available for the formation of free radical species^10^. Intravenous iron may lead to transient NTBI generation as iron is released from its carbohydrate complex too rapidly causing transferrin to become saturated (transferrin saturation > 65%), especially with second-generation IV iron compounds (e.g. iron sucrose)^11,12^. Previous in vitro studies have shown rapid release of NTBI with iron sucrose, with clinical studies linking this to the development or worsening of proteinuria^13,14^. Recently Garbowski and colleagues has suggested two mechanisms of NTBI release—a rapid component associated with direct release from the iron compound and a delayed component associated with iron released via macrophages^15^. Given the particular pharmacodynamics of the different preparations available in the market, older generation preparations (e.g. iron sucrose) can release NTBI bi-modally, a notion that appears to be present at a lesser extent with newer generation IV irons (ferric carboxymaltose > ferric derisomaltose)^15^. The generation of NTBI may lead to a cascade of effects involving labile plasma iron resulting in increased oxidative stress causing inflammation and endothelial dysfunction, which are thought to be key determinants of cardiovascular risk^16,17^. As CKD and iron deficiency represent states of increased oxidative stress and endothelial dysfunction, this is concerning^18,19^. It is therefore important to assess whether iron repletion through newer generation IV irons, which have more compact structures with lesser and slower free iron release compared to their predecessors, has any impact on these important non-traditional cardiovascular risk factors.

As a sub-study of the multicenter randomized controlled clinical trial ‘Iron & Heart’, we embarked in secondarily identifying whether treatment with a single dose of 1000 mg of FDI, a newer-generation IV iron, could adversely impact biomarkers of oxidative stress [F2-isoprostanes, thiobarbituric acid reactive substances (TBARS)], inflammation (interleukin (IL)-6, IL-8, IL-10) or endothelial function (P-selectin, E-selectin). This was performed to aid with further hypothesis generation, and identify any potential mechanistic signal of iron-induced injury.

## Results

The patient disposition consort figure has been previously published^20^. Following screening of 316 patients from the three tertiary renal centers, 54 patients were recruited in the interventional sub-study. Twenty-six patients received 1000 mg of FDI and 28 received placebo. Fifty-two patients completed all of the follow-up visits; however, not all tests were carried out in all patients nor did all patients attend every visit^21^.

The characteristics of the two arms of the interventional sub-study group have been previously published and had generally similar baseline characteristics, including mean serum creatinine (FDI: 167.0 (40.2); Placebo: 204.9 (67.3) μmol/L) and eGFR (FDI: 33.2 (9.3); Placebo: 29.1 (9.6) mL/min/1.73 m^2^)^21^. Baseline characteristics of each group (demographics, anemia and renal function), with emphasis on mechanistic data are summarized in Table 1. Supplementary Table 1 provides information on the concomitant medications of the participants at baseline.

**Table 1:** Baseline demographic, hematinic, renal function and biomarker profile of participants in study; Mean (SD) or number (%).

Characteristic	FDI	Placebo	Characteristic	FDI	Placebo
Age—years	61.6 (10.1)	57.8 (12.9)	**Hematinic values**		
			Hemoglobin—g/L	131.0 (7.4)	126.5 (11.8)
			Serum Ferritin—μg/L	64.2 (29.1)	68.4 (55.3)
			Transferrin saturation—%	22.3 (8.8)	19.7 (5.6)
**Sex**			**Renal function**		
Male	11 (42.3%)	15 (53.6%)	eGFR—mL/min/1.73 m^2^	33.2 (9.3)	29.1 (9.6)
Female	14 (53.8%)	13 (46.4%)	Serum creatinine—μmol/L	167.0 (40.2)	204.9 (67.3)
Unknown	1 (3.8%)	0 (0.0%)			
**Ethnicity**					
White	19 (73.1%)	23 (82.1%)	Markers of oxidative stress*		
Asian	1 (3.8%)	2 (7.1%)	TBARS—nM	973.7 (326.4)	799.0 (371.6)
Black	4 (15.4%)	3 (10.7%)	F2-isoprostanes—pg/mL	16,243.2 (46,770.2)	8184.0 (13,227.4)
Mixed race	1 (3.8%)	0 (0.0%)			
Unknown/other	1 (3.8%)	0 (0.0%)			
**Smoker**			Markers of inflammation**		
Current	1 (3.8%)	4 (14.3%)	IL-6—pg/mL	27.5 (40.5)	11.8 (13.1)
Previous	8 (30.8%)	9 (32.1%)	IL-10—pg/mL	54.0 (79.7)	72.7 (150.6)
No	17 (65.4%)	15 (53.6%)			
BMI—kg/m^2^	30.8 (6.8)	30.0 (6.4)	Markers of endothelial function***		
			E-selectin—ng/mL	58.8 (24.0)	50.6 (16.4)
			P-selectin—ng/mL	74.4 (64.0)	59.1 (24.7)

At baseline: FDI: N = 26; Placebo: N = 28.

*In terms of oxidative stress markers: TBARS: FDI: 22; Placebo: 24 F2-isoprostanes: FDI: 16; Placebo: 21.

**In terms of inflammatory markers: IL-6: FDI: 10; Placebo: 11 IL-10: FDI: 20; Placebo: 22.

***In terms of endothelial markers: E-selectin: FDI: 22; Placebo: 28 P-selectin: FDI: 17; Placebo: 23.

*BMI* body mass index, *FDI* ferric derisomaltose, *eGFR* estimated glomerular filtration rate, *TBARS* thiobarbituric acid reactive substances.

### Oxidative stress markers

**Figure 1:**
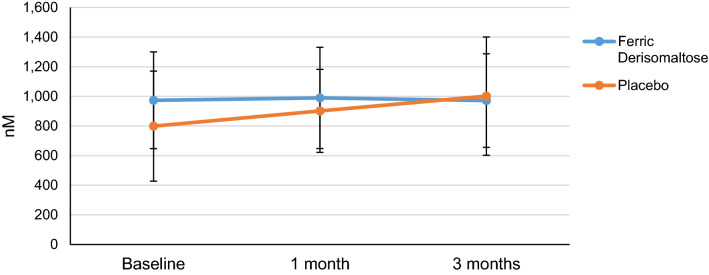
Thiobarbituric acid reactive substances (TBARS) following intervention. Legend: TBARS response following infusion with FDI or placebo. Mean values are plotted and error bars represent standard deviation. Number of samples used in the statistical analysis as per Table 2. No statistically significant difference existed between changes caused by either IV iron (Ferric derisomaltose (FDI)) or placebo as that was noted by Mann–Whitney *U* test; x-axis represents the visit points and y-axis the TBARS concentration in nM.

Oxidative stress markers with regards to mean TBARS concentration did not display any significant changes irrespective of treatment throughout the study. There was no statistically significant difference between the change in concentration in the FDI and placebo arms at any time point (1-month: *P* = 0.864; 3-months: *P* = 0.542) (Fig. 1). Mean measurements of F2-isoprostane indicated a 77% decrease 1 month following FDI infusion, compared to 66% decrease with placebo. The decreasing trend due to FDI was sustained as it remained 58% lower than the baseline value at 3 months. F2-isoprostane mean concentration increased by 23% from baseline 3 months following placebo administration. There was no statistical difference in the change in F2-isoprostane levels between the two arms (1-month: *P* = 0.758; 3-months: *P* = 0.957). Table 2 accompanies Fig. 1 delineating the samples received and analyzed per group, the results and the statistical analysis associated.

**Table 2:** Comparison of surrogate markers of oxidative stress at baseline, 1-month and 3-months following intervention. Values are presented as means with standard deviation (SD) and medians with interquartile range (IQR). N represents number of patients where data was available.

	FDI					Placebo					***P* value**
	Mean	SD	Median	IQR	N	Mean	SD	Median	IQR	**n**	
**TBARS—nM**											
Baseline	973.7	326.4	976.5	295.9	22	799.0	371.6	752.4	551.5	24	
1 month	989.4	342.0	1006.9	530.2	19	901.8	280.2	895.1	369.2	20	0.709
3 months	971.5	316.0	895.4	434.9	21	1001.6	398.9	983.7	509.1	20	0.608
**F2-isoprostanes—pg/mL**											
Baseline	16,243.2	46,770.2	3371.6	4647.9	16	8183.97	13,227.4	3358.4	6500.4	21	
1 month	3897.2	2548.6	3229.5	2911.5	14	4970.22	4337.2	3178.4	8425.9	23	0.826
3 months	6748.8	9389.9	3537.8	6432.7	19	10,043.35	25,844.5	3908.2	6146.2	21	0.303

Mann Whitney *U* was performed to compare the change in concentration. Statistical significance inferred at *P* < 0.05. N represents the number of patients where data was available.

*FDI* ferric derisomaltose, *TBARS* thiobarbituric acid reactive substances.

### Inflammation

Mean concentrations of IL-6 and IL-10 remained unchanged with FDI and placebo at both 1 and 3 months. Due to the very small sample of sizes analyzed for IL-8 no formal analysis took place. There was no statistically significant difference between FDI and placebo at baseline, 1-month and 3-months. As previously reported, FDI did not affect CRP measurements at any point in the study (FDI vs. placebo: 1-month: 4.2 (2.7) vs. 6.9 mg/L (18.3), *P* = 0.6; 3-months: 7.5 (6.8) vs. 10.3 mg/L (23.0), *P* = 0.5). Table 3 displays the number of samples analyzed per group, the results and the associated statistical analysis in terms of comparison based on change in concentration.

**Table 3:** Comparison of interleukin response at baseline, 1-month and 3-months following intervention. Values are presented as means with standard deviation (SD) and medians with interquartile range (IQR). N represents number of patients where data was available.

	FDI					Placebo					*P* value
	Mean	SD	Median	IQR	n	Mean	SD	Median	IQR	n	
**IL-6—pg/mL**											
Baseline	27.5	40.5	9.1	17.5	10	11.8	13.1	7.5	2.1	11	
1 month	31.5	46.1	7.9	36.0	8	13.1	14.3	7.4	0.7	6	0.537
3 months	30.1	43.7	8.9	13.8	10	19.5	37.7	6.9	0.7	9	0.432
**IL-10—pg/mL**											
Baseline	54.0	79.7	15.1	31.5	20	72.7	150.6	16.7	22.6	22	
1 month	55.5	77.8	17.4	36.7	19	66.9	136.9	17.2	22.3	20	0.327
3 months	52.7	80.4	14.0	28.6	21	59.8	131.5	20.4	16.3	20	0.538

Mann Whitney U was performed to compare the change in concentration. Statistical significance inferred at *P* < 0.05. N represents the number of patients where data was available.

*FDI* ferric derisomaltose, *IL-6* interleukin-6, *IL-10* interleukin-10.

### Endothelial function

P-selectin was unaffected by the FDI infusion. FDI treatment led to a significant increase in E-selectin compared to placebo at 1 (*P* = 0.003) and 3 months (*P* = 0.002) from baseline. Table 4 indicates the number of samples analysed per group, the results and the associated statistical analysis.

**Table 4:** Comparison of markers endothelial function on baseline, 1-month and 3-months following intervention. Values are presented as means with standard deviation (SD) and medians with interquartile range (IQR). N represents number of patients where data was available.

	FDI					Placebo					*P* value
	Mean	SD	Median	IQR	n	Mean	SD	Median	IQR	n	
**E-selectin—ng/mL**											
Baseline	58.8	24.0	56.1	30.7	22	50.6	16.4	45.9	22.1	28	
1 month	61.6	21.0	64.9	37.5	22	49.3	18.8	49.1	26.0	26	0.030
3 months	72.9	58.8	57.5	29.6	18	60.3	24.3	57.4	31.6	20	0.002
**P-selectin—ng/mL**											
Baseline	74.4	64.0	60.3	40.9	17	59.1	24.7	51.8	26.5	23	
1 month	80.1	76.9	61.8	37.1	17	70.2	27.1	68.9	47.3	21	0.735
3 months	58.8	24.0	56.1	30.7	22	50.6	16.4	45.9	22.1	28	0.759

Mann Whitney U was performed to compare the change in concentration. Statistical significance inferred at *P* < 0.05. N represents the number of patients where data was available.

*FDI* ferric derisomaltose.

## Discussion

When compared to placebo, a single dose of 1000 mg of IV FDI led to a non-significant decrease in markers of oxidative stress (F2-isoprostane, TBARS), resulted in no significant changes in the interleukin profile, and caused a modest increase in E-selectin without resultant changes in any clinical markers of vascular dysfunction. This accompanied a previous demonstration of no detriment to renal function and no induction of renal injury in terms of cystatin C, creatinine, proteinuria and serum Neutrophil gelatinase-associated lipocalin^22^.

The optimal management of iron deficiency in patients with CKD is unclear with some evidence suggesting benefit with intravenous iron while others raising concerns about adverse effects^10,23^. These concerns surround issues such as oxidative stress leading to endothelial and cardiac dysfunction via lipid peroxidation, cytotoxicity and activation of monocytes^24,25^. Oxidative stress is directly linked to the amount of NTBI released following IV iron administration and this is dependent on the iron formulation used; older IV iron preparations release greater NTBI amounts compared to newer compounds^12,26^. Evidence from a randomized controlled trial including 59 ND-CKD patients (stages 3–4) indicate that older IV irons (iron sucrose, low-molecular-weight iron dextran, ferric gluconate) caused a rise in TBARS within 30 min^27^. Regarding newer IV iron compounds, in vitro studies have suggested variable induction of oxidative stress and lipid peroxidation in a human kidney cell line (FDI, FCM)^28^, but the effect of different compounds in vivo may differ and its clinical relevance is unknown. Data in ND-CKD patients relevant to FDI suggests no significant increase in oxidative stress markers irrespective of dose used and no detrimental effect in terms of side effects, inflammation and renal injury in the short term following intravenous administration^29^. Similarly in our study there was no statistically significant change in markers of oxidative stress following administration of FDI at 1 month and 3 month follow-up visits and when compared to placebo. In fact, FDI either did not affect or led to a decrease in markers of oxidative stress suggesting that alleviation of iron deficiency may actually result in an anti-oxidant effect as previously noted in a pregnant population^30^.

Evidence regarding the immunologic and inflammatory effects of IV iron use is contradictory and inconsistent, with certain studies commenting on detriment to cellular functions and others indicating improvement in markers of oxidative and inflammation. Deleterious effects on mononuclear cells through a variety of pathological mechanisms have been displayed, whilst a rise in IL-6 has been seen in hemodialysis^31^. These effects may be compound dependent, with iron sucrose linked more strongly with monocyte development, activation and function^32^. Other studies have noted no sequela following administration of IV iron^33^. In the current study, markers of inflammation in non-anemic ND-CKD patients were not affected by FDI supplementation.

Previously Kuo and colleagues have noted an increase in markers of endothelial dysfunction following infusion with iron sucrose, while evidence indicated a dose-dependent increase in intima-media thickness with iron supplementation in end-stage-renal-disease^8,34^. In our cohort, a significant rise in E-selectin was found with FDI treatment; however, its clinical relevance is unknown as FDI did not affect vascular function as measured by blood pressure and pulse wave velocity measurements^21^. E-selectin is a cell adhesion molecule belonging to the selectin family that is only expressed in the endothelium and is expressed in response to certain cytokines such as TNF-α and IL-1β. It is involved in leucocyte rolling and plays a key role in atherosclerosis, while it is also associated with chronic inflammatory conditions^35^. Previous research suggests that raised expression of adhesion molecules (such as E-selectin) is seen in chronic infections characterized by increased labile intracellular iron, potentially linking iron with the modulation of adhesion molecules^36,37^.

Animal studies suggest that in CKD the impact of increased pro-oxidative factors is negated by anti-oxidant activity while there is a documented increase in pro-oxidant activity in cardiac tissue (increase in lipid peroxidation) accompanied by a compensatory increase in anti-oxidant activity^38,39^. Indeed, in this “primed” situation there is an increase in the anti-oxidant glutathione peroxidase leading to no overall increase in oxidative stress. It is therefore possible that further alleviation of iron deficiency benefits cardiac function. The observed decrease in F2-isoprostane with FDI treatment, albeit non-significant, may represent an additional benefit to alleviation of oxidative stress as previous reviews have noted an association between F2-isoprostane and cardiovascular disease. These findings may explain the improvement in cardiac function noted in our study, similar to Toblli and colleagues^21,40,41^. Iron deficiency treatment with high-dose IV iron may lead to improved myocardial mitochondrial action and mechanics alongside an overall antioxidant effect, and this may explain the reduction in cardiovascular events witnessed in large randomized controlled trials in both hemodialysis-dependent and ND-CKD patients^5,6^.

Intravenous iron has also been associated with skeletal muscle dynamics, with evidence suggesting improved skeletal muscle energetics in heart failure patients following IV iron administration as demonstrated in a study conducted by Charles-Edwards and colleagues^42^. This may be also beneficial in patients with CKD and further studies are needed to ascertain the impact of intravenous iron on muscular function in this patient group.

There are certainly limitations to our study, including the sample size, the short-follow-up period, the lack of immediate testing of relevant biomarkers following administration, and potential confounders of oxidative stress such as smoking and antioxidant medications such as vitamin supplements. As such any causation of antioxidant effect cannot be established, and further studies are necessary`0The study was designed to assess primarily the impact of IV iron on functional status; here we report on the analysis of secondary outcomes. It is important to acknowledge that any oxidative stress associated with IV iron can be acute, therefore not identified using the time-frame of our trial, as the patients were not re-assessed within hours of administration. In previous work by Kassianides and colleagues a dose-dependent rise in oxidative stress was demonstrated with intravenous iron within 2-h, which however appears to normalize within a week, and was not associated with side-effects reported or changes in vascular investigations such as pulse wave velocity^29^. An acute effect therefore cannot be assessed, verified or discredited using our trial, but it is important to highlight that any transient changes in oxidative stress or endothelial function did not yield any negative pathological signal in terms of renal function^22^. Given the small numbers included in the study, the results should be viewed with caution. Additionally, circulating levels and not tissue levels of biomarkers were analyzed, and in certain patients samples were not obtained. As such, these results cannot be extrapolated or generalized, and a larger study is needed. The trial and indeed this sub-study was not designed or powered to detect effects on these pre-specified biomarkers acutely and was underpowered for the concomitant clinical vascular tests. Considering the topic of sample size, it is noteworthy that throughout the study participants either did not attend visits, or testing was not performed leading to further impact on the study sample. Data was missing secondary to non-attendance or assay performance. These have an impact on the generalizability of the results discussed.

Nonetheless, this study was double-blinded in nature, therefore reducing bias, and it utilized a commonly prescribed dose of FDI in the ND-CKD population (1000 mg). As such, these results highlight the lack of prolonged oxidative stress induction by high-dose IV iron alongside the potential anti-oxidant effect that iron deficiency treatment may have and add further evidence in support of a high-dose, low frequency approach to iron supplementation in patients with ND-CKD. This is especially important considering the recent findings of a real world, observational study in ND-CKD patients highlighting persistent underdosing which then leads to a need for repeat infusions^43^.

In summary, this trial within the limits of its small size showed that amongst non-anemic iron-deficient CKD patients, a single high dose of FDI (1000 mg) did not statistically affect biomarkers of oxidative stress or inflammation suggesting that high-dose IV iron in this population has potentially no adverse effects on induction of oxidative stress. The study did not investigate the potential pro-oxidant effect of IV iron immediately following administration, but no signal suggesting prolonged pro-oxidation following IV iron was witnessed, despite the dose administered. A statistically significant increase in E-selectin was noted, however this did not translate into any vascular signals during the study. A larger study will be required to confirm the mechanistic safety of IV iron longer term in CKD patients with functional or absolute iron deficiency without anemia. The potential positive impact of treatment of iron deficiency without anemia in terms of a possible antioxidant effect requires further research, given the increased use of IV iron in disease states associated with pro-oxidant milieu such as CKD.

## Methods

The “Iron and Heart” study (EudraCT number: 2014-004133-16) was carried out in accordance with Good Clinical Practice guidelines, the Declaration of Helsinki and received a favorable opinion from the Northern Regional Ethics Service (NRES) Committee Yorkshire and The Humber-Leeds East, UK), approval reference number 10/H1306/40. Study participants had all details explained to them in writing and in person before giving informed consent.

The methodology has been previously published^20^. In brief, the ‘Iron and the Heart’ Study was a multicenter randomized controlled trial designed to investigate whether a single dose of 1000 mg of IV iron given over 30 min (ferric derisomaltose (FDI); Monofer^®^—Pharmacosmos A/S | Denmark) could improve exercise capacity in comparison to placebo (100 mL 0.9% NaCl) over 1 and 3 months in non-anemic CKD patients with iron deficiency. In this prospective double-blinded explorative, multicenter study, patients with established ND-CKD stages 3b-5 and serum ferritin < 100 μg/L and/or transferrin saturation < 20% were randomized in a 1:1 ratio using an online software to IV FDI or placebo solution.

A pre-specified secondary analysis on mechanistic aspects such as oxidative stress, inflammation and endothelial function was performed through measurements at baseline, 1 and 3 months. Where biomarker analysis was necessary, two blood samples were obtained from participants and placed in ethylenediaminetetraacetic acid and serum separating tubes. These were centrifuged at 3000–3500 RPM for between 5–13 min depending on the biomarker, and the plasma and serum aliquoted into cryovials. They were initially stored locally at − 80 °C prior to transfer, over dry ice, to the laboratories of the University of Hull, U.K, where they were stored further (at − 80 °C) and analyzed for markers of oxidative stress, inflammation, and endothelial function.

### Oxidative stress

Byproducts of lipid peroxidation in the form of thiobarbituric acid reactive substances (TBARS) and F2-isoprostanes were measured using standard assays as surrogate markers of oxidative stress. F2 isoprostanes were quantified using a competitive immunoassay with colorimetric (Direct 8-iso-Prostaglandin F2a enzyme Immunoassay Kit, Assay Designs, Enzo Life Science, New York, USA), whilst TBARS were measured through derivatization using an isocratic high-performance liquid chromatography technique.

### Inflammation

C-reactive protein (CRP) (Beckman Coulter | Danaher Corporation, Brea, CA, USA) and interleukins (IL-6, IL-8 and IL-10, enzyme-linked immunosorbent assay technology: Life Technologies | Thermo Fisher Scientific, Carlsbad, CA, USA) were measured as markers of inflammation.

### Endothelial function

E-selectin (Mouse E-Selectin/CD62E Quantikine ELISA Kit, R&D Systems | Bio techne, Minneapolis, MN, USA) and P-selectin (Human CD62P ELISA Kit | Abcam, Cambridge, UK) were used as biomarkers measuring endothelial function. These, combined with pulse wave velocity (PWV) measurements (Enverdis^®^ Vascular Explorer—Enverdis GmBH Medical Solutions, Jena, Germany) provide evidence relevant to vascular and endothelial dysfunction.

### Statistical analysis

An intention-to-treat approach was performed, where all randomized participants were included in data analysis. Continuous laboratory parameters were summarized using mean (standard deviations), medians (interquartile range) and categorical data using number (%). Kolmogorov–Smirnov (KS) was used to test for normality. Mann Whitney tests were used to compare the change in concentration from baseline to 1 month and 3 months, between groups given the non-normal distribution of the data and present results as mean (standard deviation) and median (interquartile range) to overcome the skewed nature of the data. All analyses were undertaken on IBM SPSS Statistics for Windows, version 26 (IBM Corp., Armonk, N.Y., USA). A *P* value of < 0.05 was considered to indicate statistical significance. No power calculation was included as this study was exploratory in nature.

## Supplementary Information

Supplementary Table 1.

## Data Availability

The data associated with the paper are not publicly available but are available from the corresponding author on reasonable request with the relevant permissions and agreement of the Research and Development Department of the Hull University Teaching Hospitals NHS Trust that served as the sponsor for the study.
