# Supplementary material for: Analysis of oxidative stress, inflammation and endothelial function following intravenous iron in chronic kidney disease in the Iron and Heart Trial
Source: Sci Rep. 2022 Apr 27;12:6853. doi: 10.1038/s41598-022-10717-8 (PMC9046378; doi:10.1038/s41598-022-10717-8)
Supplement: Supplementary file 1 — Supplementary Table 1. [file 41598_2022_10717_MOESM1_ESM.pdf]

Analysis of oxidative stress, inflammation and endothelial function following intravenous iron in chronic kidney disease in the Iron & Heart Trial

Authors:

*Xenophon Kassianides[1] (BSc, MBBS, MRCP, PgCert (Res)) – Clinical Research Fellow and Honorary Lecturer

Prof Victoria Allgar [2] (BSc, PhD, CStat, SFHEA) – Professor of Medical Statistics and Director of Peninsula Clinical Trials Unit

Prof Iain C. Macdougall [3] – (BSc (Hons), MB, ChB, MD, FRCP) – Consultant in Renal Medicine and Professor of Clinical Nephrology

Prof Philip A. Kalra [4] - (MA (Cantab) MBBChir, MD, FRCP) – Consultant in Renal Medicine and Honorary Professor

*Prof Sunil Bhandari[1] (MBChB, MRCP, FRCPE, PhD, M Clin Edu, FHEA) – Consultant in Renal Medicine and Honorary Professor

Affiliations:

[1] Academic Renal Research Department, Hull University Teaching Hospitals NHS Trust and Hull York Medical School, Hull, United Kingdom

[2] PenCTU, Peninsula Medical School (Faculty of Heath), Plymouth, United Kingdom

[3] Department of Renal Medicine, King's College Hospital, London, United Kingdom

[4] Department of Renal Medicine, Salford Royal NHS Foundation Trust and University of Manchester, Manchester, United Kingdom

***Correspondence:** Xenophon Kassianides, Academic Renal Research Department, Hull University Teaching Hospitals NHS Trust and the Hull York Medical School, Kingston upon Hull, United Kingdom, Tel: 01482 67 4308, Fax: 01482 67 4998, E-mail: x.kassianides@nhs.net

Sunil Bhandari, Academic Renal Research Department, Hull University Teaching Hospitals NHS Trust and the Hull York Medical School, Kingston upon Hull, United Kingdom, Tel: 01482 67 4308, Fax: 01482 67 4998, E-mail: sunil.bhandari@nhs.net

Supplementary table 1: Concomitant medications at baseline for each group presents as absolute number (percentage %).

	Ferric Derisomaltose (n=26)	Placebo (n=28)
β-blocker	4 (15.4%)	10 (35.7%)
RAAS inhibitor	15 (57.7%)	20 (71.4%)
Statins	13 (50.0%)	16 (57.1%)
Anti-platelet therapy	7 (26.9%)	10 (35.7%)
Oral hypoglycemic agents	4 (15.4%)	6 (21.4%)
Insulin	5 (19.2%)	6 (21.4%)
Sodium bicarbonate	2 (7.7%)	4 (14.3%)
